# Diagnostic Measures of Disease Progression in Cattle Following Natural Infection with Bovine Leukemia Virus

**DOI:** 10.3390/pathogens10080987

**Published:** 2021-08-05

**Authors:** Holden C. Hutchinson, Vickie J. Ruggiero, Bo Norby, Kelly R. B. Sporer, Paul C. Bartlett

**Affiliations:** 1 Agricultural Research Services Research Participation Program, Oak Ridge Institute for Science and Education, Oak Ridge, TN 37830, USA; 2 College of Veterinary Medicine, Michigan State University, East Lansing, MI 48824, USA; woodsvi1@msu.edu (V.J.R.); norby@msu.edu (B.N.); bartle16@msu.edu (P.C.B.); 3 CentralStar Cooperative, East Lansing, MI 48910, USA; kelly.sporer@mycentralstar.com

**Keywords:** epidemiology, bovine leukemia virus, progression

## Abstract

This study describes the longitudinal changes in bovine leukemia virus (BLV) ELISA antibodies, proviral load (PVL), and blood lymphocyte counts (LC) observed over a 2.5-year period in naturally infected cattle. The dataset utilized was from a BLV intervention field trial on three Midwestern dairy herds. Our analysis showed ELISA false negatives were more likely to occur in cattle with low PVL and normal LC. On average, negligible changes in LC were observed during six-month intervals. Periods of lymphocytosis, defined as >10,000 lymphocytes per uL of blood, were observed in 31.5% (68/216) of BLV test-positive cattle. In BLV test-positive cows, an average increase of 2900 to 3100 proviral copies per 100,000 cells was observed during each subsequent six-month sampling interval. The difference between the minimum and maximum PVL observed for an ELISA-positive cow with 3 or more observations ranged from 0 to 115,600 copies per 100,000 cells (median: 12,900; mean: 19,200). Therefore, following the identification of ELISA-positive cattle and the assessment of PVL and LC, subsequent semiannual tests to assess disease progression may not be needed. Further work is needed to determine how available diagnostic tests can be optimized to design cost-effective testing schemes for BLV control programs.

## 1. Introduction

Bovine leukemia virus (BLV) is in the family Retroviridae and genus Deltraretrovirus and is the causative agent of enzootic bovine leukosis. As a retrovirus, the BLV RNA virus is reverse transcribed into a DNA provirus that integrates into the host genome, leading to a persistent lifelong infection. The primary target for BLV is host B-lymphocytes, although it has also been detected in other cells, such as T-lymphocytes and mammary epithelium [1,2,3]. Endemic in the U.S. cattle population, BLV is estimated to infect approximately 40% of U.S. dairy cattle [4,5,6,7,8]. A growing body of research shows that BLV negatively affects animal welfare, the profitability of the dairy industry, and perhaps human health. These impacts warrant the consideration of BLV control programs.

When the prevalence is low, BLV can be controlled through the removal or segregation of all infected cattle as was accomplished in many European countries that have achieved disease-free status [9,10]. For farms with a high prevalence, this method of control is not economically feasible; the removal of all infected cattle is cost prohibitive, and the segregation of infected cattle is often constrained by farm facilities and logistics. Therefore, an alternative intervention is to reduce the transmission of BLV until the prevalence is sufficiently low that the farm can manage to cull all infected cattle and thereby achieve eradication.

The transmission of BLV among cattle is thought to occur from the transference of BLV-infected cells, which can be found in blood, milk, colostrum, saliva, and nasal secretions [11,12,13,14]. Potential routes for the transmission of infected cells include the reuse of medical equipment (e.g., hypodermic needles, palpation sleeves), biting flies, and feeding of infected milk or colostrum [15]. Unfortunately, control of all modes of transmission is costly and labor intensive. Mixed success has been observed for controlling BLV with management interventions aimed at preventing routes of transmission. For example, conflicting results were reported by three intervention field trials implementing both single-use needles and palpation sleeves and disinfection of medical equipment [16,17,18]. Given the limitations of the aforementioned control strategies, a new approach has focused on the removal of cattle thought to be the most infectious [19,20,21]. This approach requires an understanding of the epidemiology of BLV infection, infectiousness, and disease progression in relation to the available diagnostic tests.

Infection with BLV has classically been thought to develop slowly across three progressive disease states defined by lymphocyte counts (LCs) and tumor development: 1. aleukemic (normal lymphocyte count), 2. lymphocytosis (persistently elevated lymphocyte count), and 3. lymphoma or lymphosarcoma (development of tumors) [22]. At any given time, it has been estimated that 50–60% of infected cattle are aleukemic and 30–40% are persistently lymphocytic [23]. Independent of aleukemic or persistent lymphocytosis status, less than 5% of infected cattle develop B-cell lymphoma or lymphosarcoma.

In addition to the three classic disease states, researchers have recently classified BLV-infected cattle by proviral load (PVL), expressed as proviral copies per quantity of cells or DNA [24,25]. A current theory suggests that cows with a high PVL and/or high LC are at higher risk of infecting their herd mates [16]. This theory is supported by a recent field trial in which the targeted removal of cows with high PVL and high LC was successful in reducing BLV prevalence and incidence [21]. In further support, cattle with low PVL do not appear to be at high risk of transmitting disease as BLV transmission did not occur when cattle with low PVL were introduced into negative herds [26,27].

The development of lymphocytosis is thought to result from the gradual polyclonal expansion of BLV-infected cells [23,28]. The development of high PVL has been correlated with lymphocytosis [29,30]; however, Juliarena et al. (2007) showed 40% of aleukemic cows had high PVL [24]. Recently, it has been suggested that the relative level of PVL (high versus low) is established shortly after infection and is stable over time, with minor fluctuations in PVL observed [27,31]. In an experimental infection conducted by our research team, the average peak in PVL was observed 45 days post-inoculation followed by a decline or plateau to a relatively steady state until 147 days post-inoculation in a majority of the inoculated steers [32].

In another experimental infection study by Forletti et al., the establishment of PVL was associated with the BoLA DRB3 haplotype, which has also been related to high and low PVL disease states in several cross-sectional studies [31,33,34]. Limited studies, however, have reported PVL and LC levels over time by longitudinally following naturally infected cattle [35,36,37,38].

A greater understanding of the progression to high PVL, high LC disease states is necessary given the potential for BLV control through the removal of these cattle, which are now thought to be the most infectious to herdmates. The objective of this study was to describe the longitudinal changes in BLV ELISA diagnostic results, PVL, and LC in cattle naturally infected with BLV and tested semi-annually over a 2.5-year period. More specifically, we wanted to examine the relationship between observed changes in ELISA test results and both the PVL and LC, to describe the observed fluctuations in the PVL and LC measurements over time, and to assess how changes in these measurements were interrelated.

## 2. Results

### 2.1. ELISA

Two or more ELISA tests were available for 68.9% (537/779) of cows in the study database. Consistent ELISA results were observed in 76.0% (408/537) of cows with multiple observations. At least one change in ELISA status, defined as a different ELISA test result than the previous semi-annual test, was observed for 129 cows with two or more observations. Multiple changes (2+) in ELISA status were observed for 20.1% (47/234) of cows with three or more ELISA tests. In fact, 12 cows experienced three changes and four cows experienced four changes in ELISA status.

**Figure 1:**
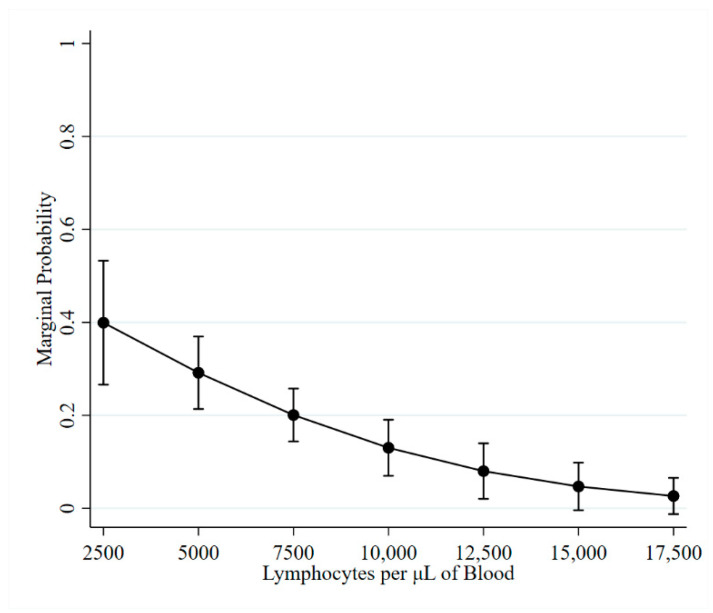
Marginal probability of experiencing a change in ELISA status as lymphocytes increase.

In separate GLMM models, increases in LC and PVL were negatively associated with the odds of experiencing a change in ELISA status. The LC for observations with a change in ELISA status ranged from 1800 to 16,600, with 75% of lymphocyte counts being less than 6700 lymphocytes per µL. The odds of a cow experiencing a change in ELISA status were reduced by 23.6% for each increase of 1000 lymphocyte per µL (*p* < 0.001). For ease of interpretation, the marginal probabilities of experiencing a change in ELISA status as lymphocytes increase are provided in Figure 1.

**Figure 2:**
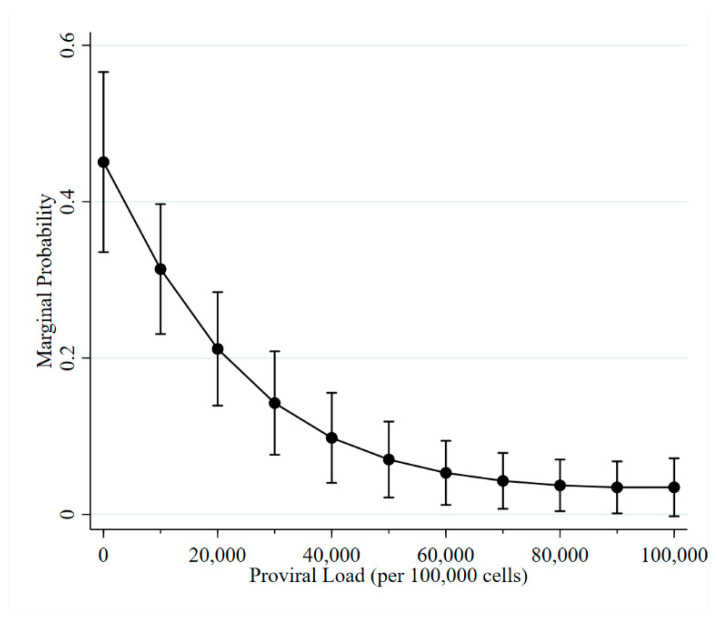
Marginal probability of a change in ELISA status as proviral load increases.

The PVL associated with a change in ELISA status ranged from 0 to 106,800 copies per 100,000 cells. However, 75% of observations had a PVL less than 5400 copies per 100,000 cells. The quadratic term for proviral load was significant. The marginal probability for a change in ELISA status is depicted in Figure 2.

Diagnostic changes to ELISA-suspect or ELISA-positive status were categorized as false positives for 12 cows and as new infections for 36 cows. Interestingly, additional changes in ELISA status were observed in 45.5% (10/22) of cows that had new infections and were subsequently retested. This change was an ELISA false negative for six cows that tested ELISA negative and PCR positive at a subsequent observation. The remaining four cows with new infections experienced changes from ELISA positive to ELISA suspect (n = 3) or suspect to positive (n = 1).

Aside from those changes that occurred following new infections, ELISA false negatives were observed in an additional 13 cows. Four cows were observed to have two ELISA false-negative results and one cow was observed to have three ELISA false negatives. Collectively, a total of 25 ELISA false-negative results, from 19 cows, were observed among 609 observations from 254 cows assumed to be BLV test positive based on combined longitudinal ELISA and PCR data. Among the 19 cows, 6 did not have subsequent tests following the false negative, 2 had consecutive false negatives with no subsequent ELISA tests, 7 had one or more ELISA-suspect or positive results after, and 4 had multiple ELISA-negatives with an ELISA-suspect or positive test in between or after the false-negative result(s). The optical density of ELISA false-negative results ranged from 0 to 0.10. The occurrence of ELISA false negatives was not associated with herd (Fisher’s Exact *p* = 0.342) or the semi-annual test (Fisher’s Exact *p* = 0.486).

**Figure 3:**
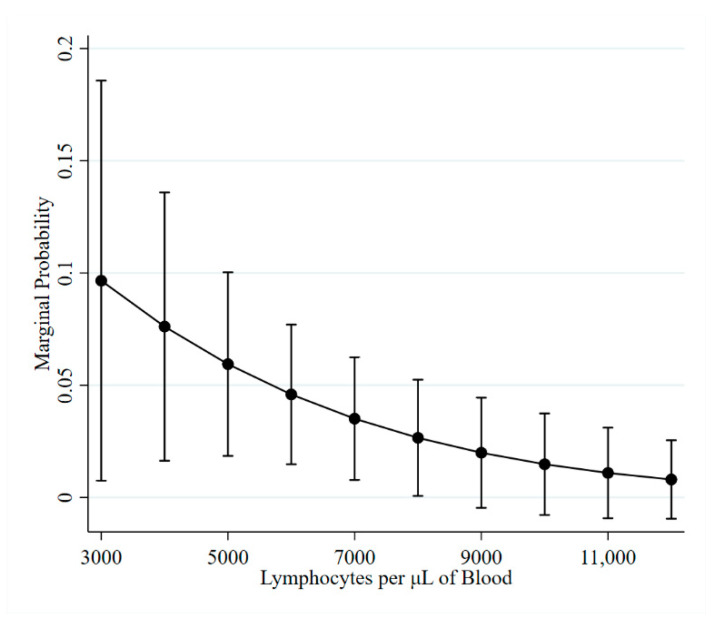
Marginal probability of experiencing an ELISA false negative as lymphocytes increase.

The median PVL associated with ELISA false-negative results was 110 proviral copies per 100,000 cells. One extreme value of 71,773 copies per 100,000 cells was associated with an ELISA false negative; the remainder of the samples had PVLs less than 1400 copies per 100,000 cells. Examining PVL results from the 13 cows that had additional testing following an ELISA false negative revealed that 12 had one or more positive PVL result. The one cow with the subsequent PVL negative result was ELISA suspect, PVL negative at the observation prior to the false negative and was PVL positive (15 copies/100,000 cells) at the time of the false-negative ELISA. Lymphocyte counts associated with ELISA false negatives ranged from 3300 to 10,200 (median: 4900) per µL of blood. A significant association was identified between lymphocyte counts and the odds of testing ELISA negative; for each increase of 1000 lymphocytes, the odds of an ELISA false negative decreased by 28.9% (*p* = 0.010; Figure 3).

### 2.2. Lymphocyte Counts

Lymphocyte counts were determined in 728 blood samples collected from 324 BLV test-positive cows. The observed LC ranged from 1800 to 23,600 lymphocytes per µL of blood (median: 6600; mean: 7700). Lymphocytosis (>7500 lymphocytes per µL of blood) was observed for 40.7% (296/728) of observations, with at least once incident of lymphocytosis observed in 51.5% (167/324) of BLV test-positive cows. Among cows with 2 or more LC observations, 49.1% (106/216) were consistently aleukemic, 30.1% (65/216) were persistently lymphocytotic, 9.3% (20/216) progressed from aleukemic to lymphocytotic, and the remaining 11.6% (25/216) were transiently lymphocytotic. When the definition of lymphocytosis was increased to 10,000 lymphocytes per µL, 23.4% (170/728) of observations were lymphocytotic and 32.7% (106/324) of cows had at least one incident of lymphocytosis. Using a cutoff of 10,000 lymphocyte per µL, 68.5% (148/216) of cows with 2 or more observations were aleukemic, 13.9% (30/216) were persistently lymphocytotic, 9.3% (20/216) progressed from aleukemic to lymphocytotic, and 8.3% (18/216) were transiently lymphocytotic.

Marginal means, which were from a model adjusted for repeated measures, were used to compare lymphocyte counts across ELISA result categories. Marginal LCs were higher among observations with ELISA-positive results (8100 lymphocytes per µL) than ELISA-suspect results (7300 lymphocytes per µL) (*p* = 0.008).

**Figure 4:**
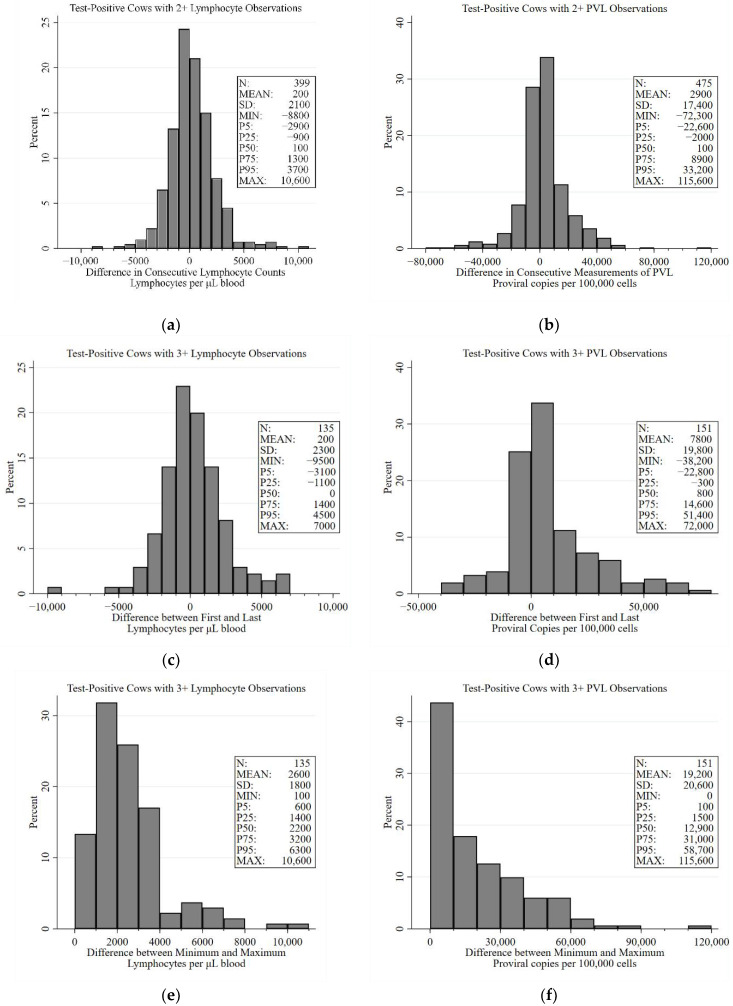
Distributions of consecutive differences, overall differences, and amplitude of lymphocytes and proviral load in BLV test-positive cattle. The consecutive difference graphs show the relative difference in lymphocytes (**a**) and proviral load (**b**) between two consecutive observations in test-positive cows. The graphs of the difference between the first and last lymphocyte (**c**) and proviral load (**d**) observation show descriptively the general trend in proviral load over time. The graphs of lymphocyte (**e**) and proviral load amplitude (**f**) show the differences between the minimum and maximum observations and indicate the absolute fluctuations in BLV test-positive cows.

Observed changes in the LC of BLV test-positive cows that occurred over an approximately six-month-long interval are presented in Figure 4a. While fluctuations upwards of 10,000 lymphocytes per µL were observed, the average change between two consecutive semi-annual tests was an increase of 200 lymphocyte per µL; 90% of all changes were between a decrease of −2900 lymphocytes per µL and an increase of 3700 lymphocytes per µL. Looking at the difference between the first and last lymphocyte count for cows with 3 or more observations, the overall difference was evenly split between increases and decreases; 90% of cows experienced changes of −3100 and 4500 lymphocytes per µL between the first and last lymphocyte counts (Figure 4c). The median amplitude (i.e., difference between the minimum and maximum lymphocyte count) for BLV test-positive cows with three or more observations was 2200 (range: 100 to 10,600) lymphocytes per µL. This amplitude was less than 3200 lymphocytes per µL in 75% of cows (Figure 4e).

In an unconditional mean model accounting for repeated measures, 81% of the variation in the observed lymphocyte counts was attributed to between-cow differences. In an unconditional growth model accounting for repeated measures, only 1% of within-cow variation was accounted for by the inclusion of time as a random slope. A non-significant (*p* = 0.877) marginal increase of 11 (95% CI: −100 to 200) lymphocytes per µL was observed for each subsequent cross-sectional sampling for BLV test-positive cows with three or more leukocyte counts (n = 135). Addition of ELISA status to the model was marginally non-significant (*p* = 0.085).

### 2.3. Proviral Load

Proviral load was measured in DNA extracted from 850 blood samples from 359 BLV test-positive cows. The observed PVL ranged from 0 to almost 180,000 provirus copies per 100,000 cells and was right skewed. Not accounting for repeated measures, PVL was significantly higher in ELISA-positive samples (median = 43,000 copies; mean = 46,100 copies; range: 0 to 179,900 copies per 100,000 cells) than in ELISA-suspect samples (median = 180 copies; mean = 10,400 copies; range: 0 to 136,700 copies per 100,000 cells) (Kruskal-Wallis *p* < 0.001). This was confirmed using an LMM accounting for repeated measures.

The average change in proviral copies between consecutive cross-sectional samplings was an increase of 2900 copies per 100,000 cells (range: −72,000 to 115,600 copies per 100,000 cells) over the approximately 6-month period (Figure 4b). The median difference between the first and last PVL observation for cows with 3 or more observations was 800 proviral copies per 100,000 cells (mean: 7800 copies per 100,000 cells). While the range of the difference between first and last observation was −38,200 to 72,000 copies per 100,000 cells, the interquartile range was smaller and ranged from −300 to 14,600 copies per 100,000 cells (Figure 4d). Because it has been suggested that proviral load undulates around a relatively steady state [27,31,32,35], the PVL amplitude was calculated. The mean maximum change in PVL was 19,200 copies per 100,000 cells (median: 12,900; range: 0 to 115,600 copies per 100,000 cells; Figure 4f). The Pearson correlation coefficient between PVL amplitude and average PVL for cows with 3 or more observations was 0.673 (*p* < 0.001).

An unconditional mean model was utilized to partition the observed variation in PVL for cows with 3 or more observations, where 7.6% of variation can be attributed to inter-herd differences while 79.5% is attributed to cow-level differences. The addition of semi-annual test number (i.e., time) as both a fixed and random effect accounted for 19.2% of the cow-level variation. A marginal increase of 3072 proviral copies per 100,000 cells was observed with each subsequent 6-month sampling (*p* = 0.001).

Independently, ELISA status and lymphocytes per µL of blood were included as predictors in the model that contained time as both fixed and random effects. Overall marginal differences in proviral copies were observed when ELISA status was added to the growth model (*p* = 0.023). Marginal differences in PVL were not statistically significant between ELISA-suspect and either ELISA-negative (5754 copies per 100,000 cells; *p* = 0.361) or ELISA-positive (4599 copies per 100,000 cells; *p* = 0.120) observations. Differences between ELISA positive and ELISA negative, however, were significant (*p* = 0.033), with a marginal increase of approximately 10,400 copies per 100,000 cells. An increase of 11 proviral copies was observed for every increase of 1000 lymphocytes per µL of blood (*p* < 0.001). Similar effects were observed in a multivariable model containing sampling number (*p* = 0.004), ELISA status (*p* = 0.021), and lymphocyte count (*p* < 0.001).

## 3. Discussion

One of our group’s research priorities was to design cost-effective control programs that cattle producers can successfully implement to reduce BLV incidence, and in turn reduce BLV prevalence and economic impact. A control program that shows promise is the selective removal of infected cattle with high PVL [21,39]. Our research results suggest that the change in PVL and LC over time is slower than originally thought, and therefore semi-annual herd testing may be more frequent than is necessary.

Proviral load testing was not commercially available at the time of this study. PVL testing is now available and currently costs $10 USD compared to $6 USD for ELISA tests (CentralStar Cooperative, Lansing, MI, USA). PVL testing can be time and labor intensive because it requires the collection of blood samples as compared with milk samples commonly used for ELISA testing. Therefore, control programs typically use milk ELISA testing to screen for BLV and conduct follow-up PVL testing of animals that are ELISA suspect or ELISA positive. Using this sampling scheme, cattle are identified as ELISA suspect or positive as a pre-condition for PVL assays to be performed; therefore, the occurrence of BLV-infected cattle having ELISA false negatives can cause confusion in the operation of a control program. Under typical control program protocols, once a cow is identified as being infected with BLV, it may be tested multiple times over the course of the intervention program to quantify proviral load. Although infection with BLV is known to result in a lifelong infection, given the viral integration into the host genome, a greater understanding of the progression of BLV infection over time and of how proviral load and lymphocyte counts change was needed. This understanding can augment control programs by reducing the frequency, and therefore cost, of repeated samplings.

With regard to ELISA tests, we were interested in the cows that had changes in ELISA status, especially going from positive to negative, and their associated proviral load and lymphocyte counts. The ELISA assay was categorized as negative, suspect, or positive based on a numerical optical density (OD) reading. The general purpose of the suspect category was to express caution in the test result because residual milk carryover (milk from the previous cow tested) may cause false positives [40]. However, in this study, 85% (131/154) of ELISA-suspect observations were PVL positive. While the OD is not the equivalent of antibody titer, it may be representative of the relative abundance of antibodies or may be associated with other BLV impacts and disease measurements. For example, a previous analysis conducted by our research team found significant differences in both milk production [41] and in the likelihood of being culled [42] based on BLV-ELISA OD categorization.

In this study, the true frequency of ELISA false negatives (i.e., ELISA negative, PVL positive cows) was unknown given the way ELISA was used as a screening test. However, because PVL was evaluated for cattle that tested ELISA negative but had a previous test at which they were ELISA suspect, ELISA positive, or PVL positive (i.e., BLV test positive), we observed that 7.4% (19/258) of cows known to previously be BLV test positive had ELISA false negatives. This proportion is similar to another study, which reported 6.8% (5/73) of cows were serologically negative and PCR positive [43]. The observance of ELISA false negatives in this study may partially be explained by the imperfect sensitivity of the milk ELISA, which was estimated to be 86% when compared to the serum-based ELISA. Interestingly, of the 19 ELISA false-negative cows, 5 (26.3%) were ELISA false negative at multiple time points. This suggests that there may be more PVL-positive, ELISA-negative cows among the 236 cows in the study database that consistently tested ELISA negative and were never tested by PCR.

We observed that both the probability of having a change in ELISA and the probability of an ELISA false negative was inversely associated with both the proviral load and lymphocyte count. The observed ELISA false negative with a high PVL (71,733 copies per 100,000 cells) may reflect a diagnostic testing error. In a study by Chen et al., PVL was not examined, but the identification of viral RNA was reported and was associated with elevated lymphocyte counts [44]. Given that lymphocyte counts are correlated with PVL [29], it is plausible that cows with low PVL and low LC may experience less viral reactivation, and thus reduced immune stimulation and antibody production. This theory is further supported by our observation that PVL was significantly lower in ELISA-suspect observations compared to ELISA-positive observations.

The cows with changes in ELISA status, specifically going from ELISA positive to negative, do not likely pose a great threat to control programs that focus on the removal of cattle with high PVL and LC. In the long term, however, the small subset of cows with repeated ELISA false negatives could prove problematic for BLV eradication programs that rely on serology screening by milk ELISA to initially identify infected cattle.

Regarding both the LC and PVL, the main outcome of interest was the observed changes over time. We sought to measure the extent to which LC and PVL increase over time in BLV-infected cows and to determine if the presence of cows with high PVL and lymphocytosis were the result of a gradual disease progression.

In this analysis, negligible increases in lymphocytes were typically observed between sequential tests conducted at approximately 6-month intervals. The addition of time into a linear growth model only accounted for a small (1%) portion of within-cow variability. Combined with the observed changes in LC, these results suggest that fluctuations in LC are not the result of gradual disease progression. Given that diagnostic reference intervals for LC typically span an approximate range of 5000 lymphocytes (2500 to 7500 lymphocytes per µL) [45], the observed fluctuations may represent normal physiological changes. There was, however, a subset of cows that experienced absolute changes in LC greater than 11,000 lymphocytes per µL during the observed study period. Whether these changes were associated with BLV disease progression or were the result of other physiological processes, such as infection with another pathogen, could not be determined.

Similar to the lymphocyte counts, changes in PVL were observed between sequential six-month sampling intervals and between the first and last cross-sectional observation. Absolute fluctuations averaged 19,200 proviral copies per 100,000 cells. In an experimental infection study, we observed that PVL typically stabilized, with minor fluctuations after an initial peak following infection [32]. Thus, it is plausible that the observed variation is just normal variation around a relatively consistent proviral load. Unlike lymphocyte counts, however, a statistically significant increase of 3072 proviral copies per 100,000 cells was observed per each six-month observation period in the LMM. Furthermore, addition of sampling number to represent time in the statistical model accounted for 19% of the within-cow variability.

The current thought is that BLV persists through the mitotic replication of infected cells. Our observation of a non-significant increase in LC coupled with a small, yet significant, increase in PVL supports the idea that infected cells survive longer and accumulate more than non-infected cells [46] or suggests that new cells are becoming infected, which may occur during periods of viral reactivation. While significant increases in PVL over time were observed, the difference between the first and last observation for cows with 3 or more observations was less than 15,000 proviral copies per 100,000 cells for over 75% of BLV test-positive cows. Given the sampling scheme, 3 or more observations corresponds to a 1.5-year period. This indicates that large increases in PVL over time do not occur for the majority of infected cattle.

Collectively, our observations indicate that, while variations are observed, the majority of BLV test-positive cows experience minor increases in LC and PVL over time. Once cattle are identified as being infected, and the LC and PVL are quantified, repeated testing as often as 6-month intervals may not be necessary. For ongoing control programs, annual BLV testing may be sufficient. While random intercepts and slopes were included in models of lymphocytes and proviral load, linear mixed models borrow analytical power collectively from all observations, and therefore do not perfectly fit the observed measurements. Thus, it is possible that while the statistical models indicated cows experience negligible to minor increases in LC and PVL with time, there is a subset of cows in which LC and PVL rapidly increase. Although reducing the sampling frequency could fail to identify these cattle, a balance must be identified that allows for cost-effective broad implementation of BLV control programs. Future studies should compare the effectiveness of intervention programs with varying sampling frequency to significantly reduce both the herd BLV prevalence and incidence.

Utilizing an existing dataset generated from an intervention field trial leads to limitations for this analysis. Given the series testing design implemented in the field trial, cows needed to have at least one ELISA test with either suspect or positive results for both PVL and LC tests to be performed. Thus, the subset of ELISA-negative cows testing PVL positive (i.e., false-negative ELISAs) or having lymphocytosis may be greater than reported. Additionally, the intervention field trial focused on the removal of cows with high proviral loads and/or lymphocytosis, which were the disease states of greatest interest. Therefore, our results may be biased because the likelihood of having longitudinal observations on these cattle was reduced. Lastly, the length of infection was unknown for cattle in the dataset, hindering the ability to associate changes in diagnostic measurements with length of infection, which could potentially provide better insight to the rate of natural disease progression.

## 4. Materials and Methods

### 4.1. Study Design

**Figure 5:**
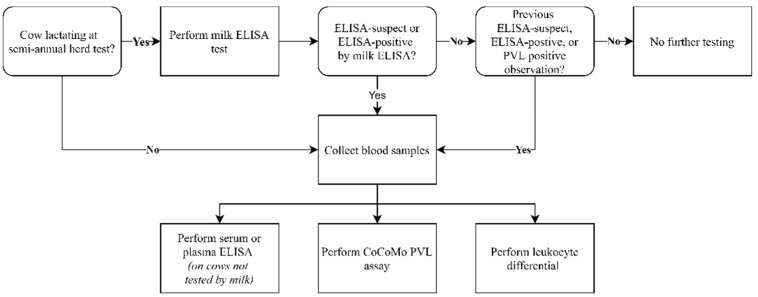
Semi-annual sampling scheme.

The data used for this analysis were from a previously published field trial, which aimed to reduce the prevalence and incidence of BLV using PVL and LC measurements as indicators of the infection potential of cows that could then be targeted for culling or for segregation until culling at a later date [21]. Three midwestern dairy herds were enrolled and sampled biannually over the course of 2.5 years following the methodology presented in Figure 5. At each semi-annual sampling, all cows that were lactating were tested by BLV antibody-capture milk ELISA. Subsequent on-farm sampling was conducted within one month to collect blood samples for PVL and LC testing from cattle that had ever had an ELISA-suspect or ELISA-positive result. In addition, blood samples were collected from cattle that were in the milking herd but were not lactating when semi-annual milk samples were collected for ELISA testing using either plasma or serum. The current study looks at the longitudinal aspects of the resulting database. A total of 779 cows were tested and contributed 2058 sampling observations. The average number of observations per cow was 2.6 (range: 1 to 5). The relative frequencies of total observations, ELISA, PVL, LC tests as well as the frequency of positive outcomes are presented in Table 1 This analysis only included observations from cows that had two or more timepoints at which they had either ELISA or PVL results indicating infection with BLV.

### 4.2. Sample Analysis

Milk and serum antibody ELISA testing were performed by CentralStar Cooperative (Lansing, MI, USA) using a modified commercial gp51 antibody capture ELISA test (IDEXX Laboratories, Inc., Westbrook ME, USA) as previously described [21,47]. ELISA reaction times were standardized to positive controls and test results were reported as corrected optical densities. Results of all ELISA tests were categorized as negative, suspect, or positive.

Blood leukocyte differentials were performed using an early version of the QScout blood leukocyte differential machine (Advanced Animal Diagnostics, Morrisville, NC, USA) as previously described [21]. The differential included the total leukocytes, lymphocytes, neutrophils, and their relative percentages, which were output directly from the QScout system. Leukocyte differentials were not performed at the first cross-sectional sampling.

Proviral load was quantified using the CoCoMo qPCR method (RIKEN Genesis, Tokyo, Japan) [48] as previously described [21]. Proviral copies were standardized to the number of nucleated cells through quantification of the BoLA-DRA gene and were reported as proviral copies per 100,000 cells.

### 4.3. Data Analysis

All descriptive and analytical statistics were performed using Stata 15 (StataCorp LLC, College Station, TX, USA).

#### 4.3.1. Descriptive Analysis

A change in ELISA status was defined as a cow having different ELISA test results (i.e., negative, suspect, positive) reported at consecutive semi-annual sample times. Changes in ELISA status were attributed to new infections, false negatives, and false positives based on the criteria presented in Table 2. All statements must be true for a change to be defined.

Only the observations from cattle classified as BLV “test positive” by combined ELISA and PCR results contributed to the descriptive distributions of both PVL and LC. In evaluating the LC of BLV test-positive cows, persistent lymphocytosis was defined by all observed LC being greater than the specified cutoff; transient lymphocytosis was defined by the observance of both normal and lymphocytic results over time in a BLV test-positive cow. Cut-offs of both 7500 and 10,000 lymphocytes per µL of blood were examined and used to define lymphocytosis based on diagnostic laboratory reference intervals and BLV-related literature, respectively [23,45].

#### 4.3.2. Analytical Analysis

A generalized linear mixed model (GLMM) approach was used to examine how PVL and LC affected changes in ELISA status in BLV test-positive cattle across semi-annual sampling points. Using a binomial distribution and a logit link, two separate outcomes were examined: any change in ELISA status and a change associated with an ELISA false negative. The linearity of continuous variables in the logit was assessed using the Box-Tidwell statistic. The low frequency of false negatives resulted in categorical variables with zero counts that could not be included in bivariable logistic regression models because of perfect prediction. Therefore, the associations between categorical predictors and ELISA false negatives were assessed using Fisher’s exact tests.

Changes in LC and PVL over time in BLV test-positive cattle were evaluated using linear mixed models (LMMs). Models of PVL included random effects at both the herd and cow level. The PCR assay number was included as a random crossed effect in PVL models to account for systematic variation in proviral load that may have been the result of inter-plate variability. Potential explanatory variables in PVL models were LC and ELISA status. Models of LC included a random effect for each cow. The standard errors of herd-level random effects could not be estimated in the unconditional lymphocyte model and, therefore, herd was not included as a random effect. Only ELISA status was included as an explanatory variable in LC models. In addition, linear growth models were created by including the semi-annual test number as both a fixed and random effect slope to estimate both the average and individual change over time. Only BLV test-positive cows with three or more outcome measurements were included in the linear growth models. All LMMs were built using restricted maximum likelihood estimation using Kenward-Rodger adjustment for degrees of freedom. The normality of residuals was visually assessed by histograms and Q-Q plots. Levene’s test was used to evaluate the homogeneity of variance across categorical predictors. Wald’s test was used to assess the statistical significance of independent variables. The statistical significance of marginal comparisons was adjusted using the Bonferroni correction for multiple comparisons.

## 5. Conclusions

In this analysis, we identified that a small portion of ELISA-positive cattle became ELISA negative when re-tested at 6-month sampling periods. These cattle tended to have low proviral loads and normal lymphocyte counts, and therefore likely pose little transmission risk to herd mates. Examination of the longitudinal changes in LC and PVL identified that, although both measures varied over time, most cows do not experience large changes that may be indicative of disease progression or an increase in their potential to infect susceptible herd mates. Confirmation is needed, but our results suggest that PVL and/or LC testing at 6-month intervals may be more frequent than necessary for most disease control programs.

## Figures and Tables

**Table 1:** Cow-level observation, ELISA, proviral load, and leukocyte differential test frequencies (n = 779 cows).

	Total Number of Observations					
	0 ^a^	1	2	3	4	5
Observations Overall	--	29.8%	21.1%	18.0%	17.6%	13.6%
		(232/779)	(164/779)	(140/779)	(137/779)	(106/779)
ELISA Tests	0.4%	30.7%	20.6%	18.2%	17.2%	12.8%
	(3/779)	(239/779)	(161/779)	(142/779)	(134/779)	(100/779)
ELISA-SP ^b^	47.9%	12.2%	10.9%	10.8%	10.4%	7.8%
	(373/779)	(95/779)	(85/779)	(84/779)	(81/779)	(61/779)
PVL Tests	47.1%	16.6%	14.3%	11.4%	9.0%	1.7%
	(367/779)	(129/779)	(111/779)	(89/779)	(70/779)	(13/779)
PVL-Positive	51.2%	16.8%	13.0%	10.4%	7.1%	1.5%
	(399/779)	(131/779)	(101/779)	(81/779)	(55/779)	(12/779)
Leukocyte Differentials	52.5%	16.2%	11.3%	11.2%	8.9%	-- ^c^
	(409/779)	(126/779)	(88/779)	(87/779)	(69/779)	
Combined ELISA, PVL, LC	53.5%	16.7%	11.3%	10.9%	7.6%	-- ^c^
	(417/779)	(130/779)	(88/779)	(85/779)	(59/779)	

^a^: Reflects the percentage of cows that did not have the respective test or results; ^b^: ELISA-SP = ELISA-suspect or ELISA-positive outcome. ^c^: Leukocyte differentials were not performed at the first semi-annual test.

**Table 2:** Required criteria † for changes in ELISA to be defined as new infections, false-negatives, and false-positives.

ELISA New Infection
ELISA-suspect or ELISA-positive result
Concurrent or subsequent PVL-positive result(s)
Prior ELISA-negative result(s)
No Prior PVL-positive result(s)
**ELISA False Negative**
ELISA-negative result
Prior ELISA-suspect or ELISA-positive result Either concurrent, or prior and subsequent PVL-positive test(s)
**ELISA False Positive**
ELISA-suspect or ELISA-positive result
Concurrent PVL-negative result
Prior or subsequent ELISA-negative result(s)
Prior or subsequent PVL-negative result(s)

† All statements must be true for a change to be defined.

## Data Availability

Data is available upon request.
